# Batch-Mode Analysis of Thermophilic Methanogenic Microbial Community Changes in the Overacidification Stage in Beverage Waste Treatment

**DOI:** 10.3390/ijerph17207514

**Published:** 2020-10-15

**Authors:** Shuhei Matsuda, Takahiro Yamato, Yoshiyuki Mochizuki, Yoshinori Sekiguchi, Takashi Ohtsuki

**Affiliations:** 1 Graduate School of Medicine, Engineering and Agricultural Sciences, University of Yamanashi, Kofu, Yamanashi 400-8510, Japan; g17dhl03@yamanashi.ac.jp (S.M.); tohtsuki2@gmail.com (T.Y.); 2 Iwata Chemical Co. Ltd., Iwata, Shizuoka 438-0078, Japan; ymochiduki@i-kagaku.co.jp (Y.M.); ysekiguchi@i-kagaku.co.jp (Y.S.)

**Keywords:** batch culture, beverage waste, methanogenic microbial community, overacidification

## Abstract

Biogasification by methane fermentation is an important and effective way to utilize beverage wastes. Beverage wastes are good feedstocks for methane fermentation because of their richness in sugars and proteins, although overacidification and inhibition of methane production caused by high substrate loading often become problematic. This study investigated changes in microbial communities in the overacidification state of the thermophilic methane fermentation process with beverage waste by establishing a simulated batch culture. We assessed 20 mL-scale batch cultures using a simulant beverage waste mixture (SBWM) with different amounts of addition; high cumulative methane production was achieved by adding 5 mL of SBWM (11358 mg—chemical oxygen demand—COD/L of organic loading), and overacidification was observed by adding 10 mL of SBWM (22715 mg—COD/L of organic loading). The results of 16S rRNA amplicon sequence analysis using nanopore sequencer suggested that *Coprothermobacter proteolyticus*, *Defluviitoga tunisiensis*, *Acetomicrobium mobile*, and *Thermosediminibacter oceani* were predominantly involved in hydrolysis/acidogenesis/acetogenesis processes, whereas *Methanothrix soehngenii* was the major acetotrophic methane producer. A comparison of microbial population between the methane-producing cultures and overacidification cultures revealed characteristic population changes especially in some minor species under 0.2% of population. We concluded that careful monitoring of population changes of the minor species is a potential indicator for prediction of overacidification.

## 1. Introduction

Methane fermentation is a conventional biological process that produces methane from various organic wastes. Methane fermentation has recently attracted attention as an efficient bioenergy production method to tackle global environmental problems associated with the use of fossil fuels [1].

Many studies of methanogenic microbial community dynamics in methane fermentation have been conducted using continuous or semi-continuous culture. Of course, methane fermentation is usually continuous process and its study of microbial community dynamics is important. However, stepwise changes of experimental conditions in a continuous culture preclude the comparison of initial response to each condition among microbial community at a ‘same state’ as represented by population of members. In small-scale batch culture experiment, by starting multiple cultures from one inoculum culture, it is possible to make the initial states of microbial communities constant, and to compare the response of the communities to different conditions. In our knowledge, there is no report to investigate dynamics of methanogenic microbial community in batch culture, though studies to assess correlation between methane production and experimental conditions have been conducted with batch culture [2,3,4,5].

In food waste, the amount of beverage waste is enormous; for example, the United Kingdom was estimated to generate 1,300,000 tons of beverage waste in 2012 [6]. In Japan, 2,232,000 tons (this may include solid waste) were estimated as wastes from beverage-related business in 2017 [7]. To our knowledge, there is only a countable number of studies for stable production of methane from beverage waste, although the importance of treatment has been recognized [8,9]. Additionally, the existing reports described mesophilic methanogenic processes, and thus the study of thermophilic process has been desired.

The methanogenic microbial community comprises countless microorganisms, and the process of methane production is highly complicated. In general, under anaerobic conditions, eubacteria perform substrate hydrolysis, acid production (acidogenesis), hydrogen and carbon dioxide production, and methanogens produce methane from acetate, formate, hydrogen, and carbon dioxide (methanogenesis) [10]. Therefore, acetate produced during the acid production process (acetogenesis) is a particularly important metabolic intermediate. In the acidogenesis process, volatile fatty acids (VFA), such as acetate, butyrate, and propionate, are produced, but methanogens cannot directly use VFA other than acetate and formate. The production of acetate by the oxidation of propionate or butyrate is an endergonic reaction, and if not maintained at an appropriate concentration, the function of the fermenter will be reduced [11].

In methane fermentation, challenges to increase the amount of substrate input per unit time have affected efficient production of methane. However, if the substrate load is high, VFA accumulate and methane production is inhibited [12]. This phenomenon is known as overacidification status. For stable methane production, considerable researches investigating the relationship between methanogenic microbial community dynamics and VFA accumulation have been conducted with food wastes and manures as feedstocks [13,14,15,16,17]. In the previous reports, no indication generally observed in changes of microbial communities has been found in the phylum or family level, while some parameters such as changes of propionate/acetate ratio have been proposed as signs of instability of methanogenic process [18]. To obtain the detailed information of microbial community dynamics, a trial to compare the methanogenic community members between stable and instable states in the ‘species’ level has been needed.

This study aimed to investigate the difference of initial response of thermophilic microbial community to different organic loading by small-scale batch culture with the stably maintained sludge as a ‘same state’ inoculum. In this study, we compared the behavior of microorganisms in the methane-producing state and in the overacidification state under different substrate loading conditions. Furthermore, possible indicator microorganisms in the species level for the prediction of overacidification occurrence are suggested.

## 2. Materials and Methods

### 2.1. Maintenance of Anaerobic Sludge

Anaerobic sludge was collected from a 1000-kL digester of a waste beverage treatment plant located in Shizuoka, Japan. The 3-L volume sludge was initially purged with nitrogen gas and anaerobically maintained in a fermenter (Model BMS-05PI, Biott, Tokyo, Japan) at 55 °C with agitation at 100 rpm. Simulant beverage waste mixture (SBWM) was continuously fed at a rate of 50 mL/day (757 mg-chemical oxygen demand—COD/L day of organic loading rate) and simultaneously, the wastewater was drained at the same rate (HRT 60). The SBWM used for this study included (*v*/*v*): 40% 1/2 MRS broth (Oxoid, Hampshire, UK), 20% coffee (UCC Holdings, Hyogo, Japan), 10% orange juice (Ehime Beverage, Ehime, Japan), 10% isotonic drink (Coca-Cola Customer Marketing Company, Tokyo, Japan), 5% green tea (Lifedrink Company, Osaka, Japan), 5% black tea (Kirin Beverage Company, Tokyo, Japan), 5% soymilk (Kikkoman, Chiba, Japan), and 5% yogurt (Morinaga Milk Industry, Tokyo, Japan). The ratio of SBWM was determined based on the feedstock being treated at the plant where the inoculation source was provided. The pH was adjusted to 7.0 with sodium hydroxide, and the mixture was autoclaved. Twelve month-cultured sludge with stable production of 0.6 L methane/L day was used as an inoculum.

### 2.2. Methane Production at High Substrate Loading in Batch Culture Experiments

There is a thermophilic methane fermentation plant in Shizuoka, Japan, which currently use beverage waste as the main feedstock. In the plant, high substrate loading often lowers pH and decreases methane production. We succeeded in optimizing a condition where pH was decreased by high substrate loading in batch culture at 55 °C using the inocula, which acclimated for SBWM. The inocula, sterilized water, and SBWM were mixed in 30-mL glass vials. The amount of SBWM added was changed to prepare the variation of substrate loading (Table 1). Each vial was sealed with a butyl rubber stopper and plastic cap. The headspace of each glass vial was purged with nitrogen gas for 1 min and statically cultured at 55 °C for 15 days.

### 2.3. Analytical Methods

The COD of SWBM was determined using Spectroquant COD Cell Test Kit (MilliporeSigma, Burlington, MA, US). Every five days of batch culture experiment, the normalized volume of positive pressure gases in the vials were measured and collected using needles and syringes. Then, the caps of the vials were opened, and 1.5 mL of culture suspensions were sampled. After sampling, the vials were sealed, re-purged with nitrogen, and continued the culture. Methane contents in the collected gas samples were determined by gas chromatography (Model GC2014, Shimadzu, Kyoto, Japan) equipped with a Molecular Sieve 5A 60–80 column (3 mm ID × 3 m, GL science, Tokyo, Japan) and a thermal conductivity detector. The column temperature was maintained at 40 °C for 15 min, and thereafter, the temperature was increased by 20 °C/min and finally maintained at 200 °C for 20 min. The temperature of the injector and detector was 200 °C. Nitrogen was used as the carrier gas (30 mL/min). The sampled culture suspensions were centrifuged at 11,000× *g* for 7 min, and the pellets were stored at −20 °C for DNA analysis. The pH, electrical conductivity (EC), and nitrate concentration of the supernatants were measured using pocket meters (LAQUAtwin Models B-212, EC-33, and B-742, Horiba, Kyoto, respectively). Contents of VFA in supernatants were determined by gas chromatography (Model GC-2014, Shimadzu, Kyoto), which was equipped with an HP-Innowax column (0.53 mm ID × 15 m 1.0 μm thick, Agilent Technologies, Santa Clara, CA, US) and a flame ionized detector. The column temperature was maintained at 100 °C for 34 min, and thereafter, the temperature was increased by 10 °C/min and finally maintained at 220 °C for 25 min. The temperatures of the injector and detector were 250 °C and 300 °C, respectively. Helium was used as a carrier gas (3.59 mL/min, 10:1 of split ratio).

### 2.4. Amplicon Sequence Analysis of 16S rRNA Genes Using a Nanopore Sequencer

Genomic DNA was extracted from the pellets harvested from the culture as per previously described methods [19]. Ten nanograms of extracted DNA was amplified by PCR with the Tks Gflex DNA polymerase (Takara Bio, Shiga, Japan). The primer sets Pro341F (5′-CCTACGGGNBGCASCAG-3′) and Pro805R (5′-GACTACNVGGGTATCTAATCC-3′) were used [20]. PCR amplification was performed by an initial denaturation at 98 °C for 2 min, followed by 11 cycles of denaturation at 98 °C for 10 s, annealing at 65 °C for 15 s (the temperature was decreased by 1 °C every cycle to reach the temperature at 55 °C), and extension at 68 °C for 30 s; this was followed by an additional 24 cycles of denaturation at 98 °C for 10 s, annealing at 55 °C for 15 s, and extension at 68 °C for 30 s. Purification and barcoding of PCR products were performed using the PCR Barcoding Kit (SQK-PBK004, Oxford Nanopore Technologies, Oxford, UK). The AxyPrep MAG Clean-Up kit (Corning, NY, USA) was used for all purification steps of the PCR products. The concentration of genomic DNA and PCR products was checked using the Qubit dsDNA HS Assay Kit (Thermo Fisher Scientific, Waltham, MA, USA). Twelve barcoded PCR products were mixed at 8 femtomoles each and loaded on a flow cell (FLO-MIN106 R9, Oxford Nanopore Technologies, Oxford, UK); sequencing was performed using the MinION Mk1B apparatus (Oxford Nanopore Technologies, Oxford, UK).

### 2.5. Data Analysis

Sequenced data collecting, base calling, and quality checking were performed with the MinION software (Release 18.12.6, Oxford Nanopore Technologies, Oxford, UK) to obtain fastq files. FASTQ BARCODING (v.3.10.2, Oxford Nanopore Technologies, Oxford, UK) in the EPI2ME Desktop Agent (v.2.59.1896509, Oxford Nanopore Technologies, Oxford, UK) was used to sort and to remove barcode sequences.

Statistical analysis of the microbial community structure in the family or genus level was performed using Metagenome@KIN software (v.2.0.0, World Fusion, Tokyo, Japan) which based on R package (v.3.1.0, The R Foundation, Vienna, Austria). Sequences with ≥97% similarity were assigned to the same operational taxonomic units (OTU). Representative sequence for each OTU was used for taxonomic annotation with BLAST (National Center for Biotechnology Information, Bethesda, MD, USA). The 16S rRNA database release 120.0 of DNA Data Bank of Japan (released on May 29, 2020) was used for reference. Numbers of OTUs were log-transformed when necessary to normalize the distribution and to achieve homogeneity of variance. To illustrate the analysis results, ClustVis web tool [21] was also used.

In comparison of microbial population changes among the batch cultures in the species level, we illustrated ‘growth curve-like’ figure for comprehensive presentation. FASTQ WIMP (v.3.2.1, Oxford Nanopore Technologies, Oxford, UK) was used for direct assignment of fastq sequence reads (not dependent on OTU strapping) for species level monitoring. The WIMP workflow employs a novel microbial classification engine ‘Centrifuge’ that enables rapid and accurate assignment of reads and quantification of species [22].

## 3. Results and Discussion

### 3.1. Methane Production in Batch Cultures Using SBWM

**Figure 1:**
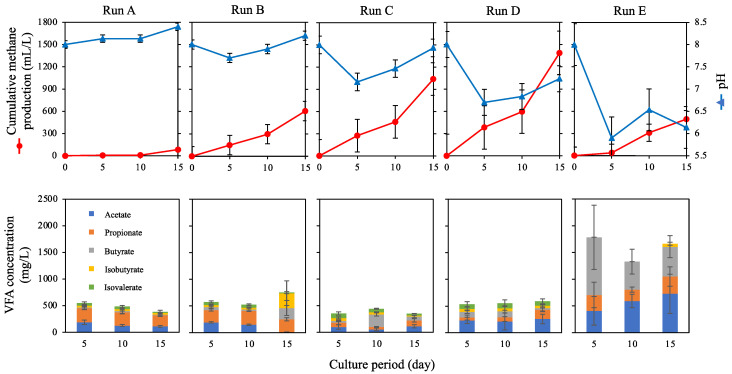
Cumulative methane production and pH changes (upper row), and concentration of VFA (lower row) in the batch cultures with simulant beverage waste mixture (SBWM). The amounts of SBWM added were 0, 0.5, 2, 5, and 10 mL for runs A, B, C, D, and E, respectively. Experiments were performed in triplicate, and error bars represent standard deviations.

We conducted batch cultures with different substrate loading. Addition of 0.5, 2, 5 and 10 mL SBWM corresponded with 1136, 4543, 11,358, and 22,715 mg-COD/L of organic loading, respectively. Cumulative methane production and changes in pH and VFA contents in batch cultures with different amounts of SBWM added are shown in Figure 1. In blank cultures without the addition of SBWM, negligible levels of methane production and pH change were observed (run A), thereby indicating that the feeding of SBWM in maintenance culture was minimal and that changes in the batch experiment could be attributed to the addition of SBWM. The highest cumulative methane production (1387.5 mL/L) was obtained with 5 mL of SBWM addition (run D), whereas production drastically decreased (493.5 mL/L) with 10 mL of SBWM addition (run E). The pH decreased at day 5 in all SBWM-added cultures. In run E, the pH decreased to 6.1 and did not return above pH 7 until day 15, although pH in the other cultures increased at days 10 and 15. The decrease in pH was considered to be mainly due to the accumulation of VFA. According to Steinbusch et al. [23], methane production decreases rapidly and butyrate production increases at pH 6. The correspondence between the drastic decrease in methane production and pH decrease with 10 mL of SBWM addition clearly demonstrated acid-inducing inhibition of methane production.

The pH decreased depending on the amount of SBWM added, and therefore we checked the VFA contents in the culture supernatants. Volatile fatty acids mainly comprised acetate, propionate, and butyrate. Propionate and butyrate have been known to be used by methanogens after conversion to acetate by eubacteria [24]. Thus, the change in VFA concentration is one of the important indicators in methane fermentation. In runs A, B, C, and D, the total VFA content was around 500 mg/L throughout the culture period. In run D, which had the highest methane production, the propionate concentration was lower than in other runs, and no other VFA accumulated. However, the total VFA amounts in runs A, B, C, and D were highly similar, and there was no correlation with the decrease in pH. In contrast, in run E, acetate, propionate, and butyrate accumulated with the highest concentrations of 725, 324, and 1080 mg/L, respectively. The amount of acetate increased with the passage of the culture time, whereas the amount of butyrate reached a maximum on the fifth day of the culture. High concentrations of butyrate are not considered to affect methane production [25]. However, Redzwan and Banks [26] reported that methane production could be inhibited by an excess of total VFA over 1500 mg/L. In run E, it was suggested that overproduction of total VFA would result in the suppression of methanogenic archaea. Accumulation of VFA and decrease of pH in run E clearly indicate that the microbial community got into the overacidification status.

**Figure 2:**
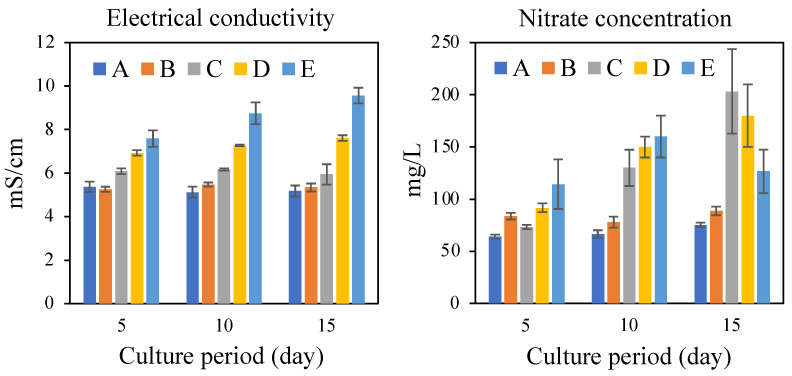
Electrical conductivities (left panel) and concentrations of nitrate (right panel) in runs A, B, C, D, and E. Experiments were performed in triplicate, and error bars represent standard deviations.

The electrical conductivity (EC) of the culture supernatant in all batch cultures increased with the amount of SBWM added (Figure 2). However, the concentration of nitrate in the culture supernatant did not behave the same way (Figure 2). This increased in a time-dependent manner to reach a peak value at day 15 in runs C and D, whereas the concentration in run E peaked at day 10 and then decreased at day 15 (Figure 2). Considering that total VFA amounts were approximately equivalent in runs A–D, except in run E, additional ionic substances other than VFA and nitrate may affect EC. Therefore, methane production in the microbial community used in this study was not suppressed by 100–200 mg/L levels of nitrate concentration experienced in runs C and D (Figure 1 and Figure 2). Methane production by methanogenic microbial communities cultured in rich media, such as rumen and feces, can be typically inhibited by the presence of 300–600 mg/L of nitrate [27,28,29]. In contrast, methane production was inhibited under oligotrophic conditions, such as in soil, at approximately 60 mg/L of nitrate [30]. In this study, although SBWM was apparently rich in organic matter, the microbial communities maintained methane production and were not suppressed by the 100–200 mg/L level of nitrate concentration.

### 3.2. Changes in the Microbial Population in Batch Cultures with SBWM

The amplicon sequence analysis of 16S rRNA genes resulted in between 212638 to 826885 quality-checked reads per sample obtained from batch cultures with SBWM. Populations of the phyla more than 1% consisted of Firmicutes (38.2–60.7%), Thermotogae (16.5–48.5%), Synergistetes (1.8–10.9%), Euryarchaeota (0.3–8.0%), Proteobacteria (0.7–2.7%), Actinobacteria (1.0–2.3%), Bacteroidetes (0.02–2.1%) and Chloroflexi (1.0–1.9%). Tyagi et al. [12] have reported that Euryarchaeota (7.6–44.4%), Proteobacteria (4.7–38.0%), Bacteroidetes (9.7–27.1%), Firmicutes (8.4–22.7%) and Synergistetes (0.7–13.2%) were the dominant phyla in the mesophilic anaerobic pulsed bed filter system treating beverage wastewater. In contrast, the microbial community in our study showed the greater abundance of Firmicutes and Thermotogae. These phyla have often been found in thermophilic methane production process [31], indicating that there were so many members involved in hydrolysis/acidogenesis/acetogenesis processes.

The Simpson and Shannon indices of microbial communities in the batch cultures were shown in Table 2. These indices reflect community diversity; the Simpson index is sensitive to community evenness, while the Shannon index is sensitive to community richness. The microbial community in run D with the highest methane production had the largest Simpson index (0.708–0.725) and Shannon index (2.248–2.369), indicating that the species richness of the community increased but some specific species became more dominantly. However, both the Simpson and Shannon indices of the microbial community in run E were apparently smaller than those in run D, meaning that the richness and evenness were decreased.

**Figure 3:**
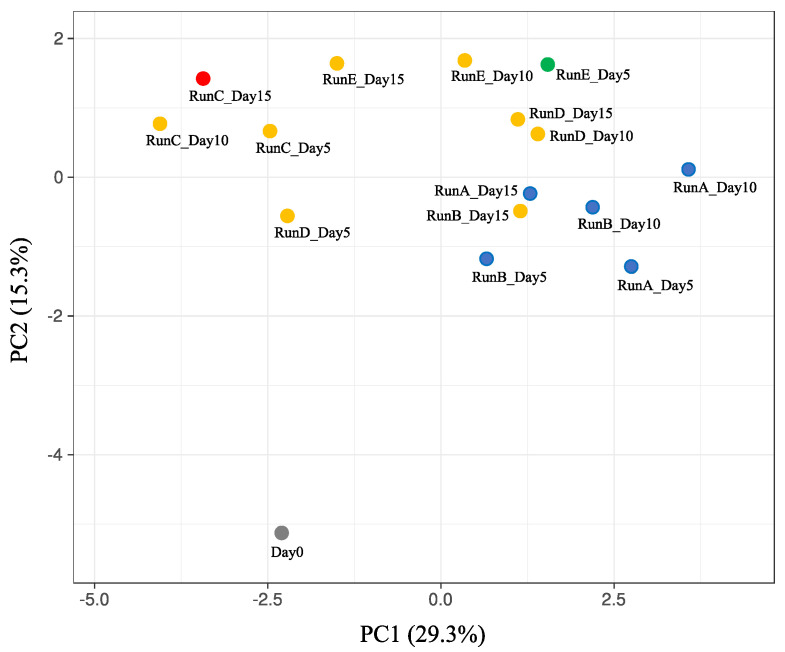
Principal component analysis of the taxonomic compositions among the batch cultures. Annotated numbers of operational taxonomic units (OTUs) were log-transformed. Unit variance scaling was applied to rows; singular value decomposition with imputation was used to calculate principal components. Samples were also clustered using correlation distance and average linkage, and the plots belonging to same clusters are indicated by same colors.

To visualize the differences of community structure, principal component analysis (PCA) and clustering of the taxonomic compositions in the family level were performed, and the results are shown in Figure 3. The plots of the communities in same runs tended to gather together, and the result of clustering demonstrated that the communities in run A and B formed a different cluster from those in other runs. Interestingly, the communities at day 15 in run C and day 5 in run E formed completely independent clusters each (Figure 3). There was a possibility that a depletion at day 15 in run C and an initial excess feeding at day 5 in run E of SBWM nutrients in the cultures would affect the diversities of community.

**Figure 4:**
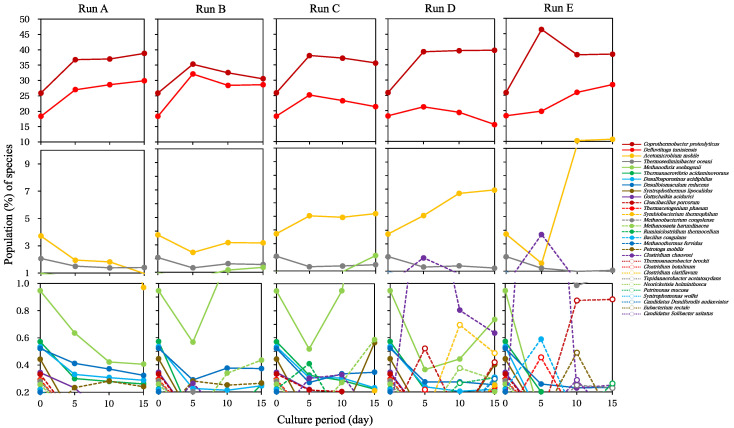
Changes in population of the species in runs **A**, **B**, **C**, **D**, and **E**. The species that amounted more than 0.2% population are shown.

Figure 4 shows the detailed changes in population of the species more than 0.2%. The predominant species, *Coprothermobacter proteolyticus* and *Defluviitoga tunisiensis*, were identified in all runs. *C. proteolyticus* is frequently found in the anaerobic digestion process [32,33] and is presumed to participate in the degradation of proteins mainly supplied with soymilk in SBWM. *D. tunisiensis* is known to assimilate carbohydrates during the anaerobic digestion process [34]. These predominant species were considered to play major roles in the production of hydrogen, carbon dioxide, and VFA, such as acetate, in batch cultures. *Thermosediminibacter oceani* constituted between 1% and 3% of the population, and acetate production by this bacterium has also been reported previously [35]. *T. oceani* exhibited stable population throughout all runs.

In runs D and E, significant changes in the population of *Acetomicrobium mobile*, *Clostridium chauvoei*, and *Methanothrix soehngenii* were observed (Figure 4D,E). An increase in the population of *A. mobile* was observed in a dose-dependent manner with SBWM addition, especially in 10% of the population on days 10 and 15 in run E. The population of *C. chauvoei* also increased by >1% on day 5 in runs D and E, although this species is generally conceived as a minor member in the anaerobic digestion process. *A. mobile* and *C. chauvoei* have been reported to produce acetate, butyrate, propionate, hydrogen, and carbon dioxide [36,37]. There was a possibility that the increase in population of these species, in addition to the predominant species *C. proteolyticus* and *D. tunisiensis*, was key to the accumulation of VFA in run E.

*M. soehngenii* is an acetotrophic methanogen [38], and its population decreased on day 5 in all runs. In runs B, C, and D, population of *M. soehngenii* recovered on days 10 and 15, but not in run E (Figure 4). The optimal pH for growth of *M. soehngenii* ranged from 7.4 to 7.8 [38], suggesting that a failure of population-recovery in run E could be correlated with the decrease in pH. The total population of *M. soehngenii* decreased from 0.94% to 0.73% in run D, and 0.072% in run E (Figure 4). However, the total population of *M. soehngenii* in run C increased to 2.1% was the highest, although cumulative methane production was lower than that in run D (Figure 1). It is already known that balanced reaction rates of hydrolysis, acidogenesis, acetogenesis, and methanogenesis are necessary for stable and efficient production of methane [39,40]. In this study, the highest cumulative production of methane was achieved in run D; however, the balance between eubacteria involved in hydrolysis/acidogenesis/acetogenesis and methanogenic archaea involved in methanogenesis seemed to be prone to breakdown.

The microbial community in run E quickly developed into a rich diverse population, which was crowded with species that each constituted less than 1% of the overall population, and the population of methanogens, including *M. soehngenii,* drastically decreased to levels lower than 0.2% (Figure 4 run E). Interestingly, the population of *Thermoanaerobacter brockii* increased on days 10 and 15. *T. brockii* has been reported to yield methane from branched-chain amino acids [41]; therefore, this bacterium may contribute to a slight accumulation of methane in run E.

From another viewpoint, we identified species with an increase or decrease in population changes more than 10-fold in all species assigned (Table 3). In methanogens, the population of *Methanosaeta harundinacea* decreased in run E only, together with the population of *M. soehngenii*. *M. harundinacea*, and *M. soehngenii* are methane producers via acetate assimilation [38,42], and the population decreases in these species could be related to the accumulation of acetate and decrease in methane production in run E. The population of *C. chuvoei* changed with 10-fold or more differences in all SBWM-added cultures (runs B, C, D, and E), whereas that of *Clostridium formicaceticum* experienced drastic changes only in run E (Table 3). *C. formicaceticum* is known to be a highly efficient acetate producer [43]; thus, this species also contributes to the accumulation of acetate in run E. Three species, *Petrimonas mucosa*, *Paludibacter propionicigenes*, and *Candidatus Solibacter usitatus*, have been found in methanogenic microbial communities as members involved in hydrolysis and acidogenesis [44,45,46]. It should be noted that *Neorickettsia helminthoeca* also experienced drastic changes in the population in runs D and E. *N. helminthoeca* and *Candidatus Solibacter* sp. have been reported to be able to solubilize inorganic phosphorus in grass-field soil [47], although the roles of these species in methanogenic microbial communities are unknown. These results were in accordance with the decrease of richness and evenness of the microbial community in run E. Even though the population levels were low except for *C. chauvoei*, there is a possibility that changes in population of species shown in Table 3 may become indices prior to overacidification in the methane production process.

In order to promote the use of waste beverages by methane fermentation in society, it is most important to establish a technology for stable production of methane. Once methane production stops due to overacidification, it is hard to recover the methane production again, leading to economic losses. Therefore, the breakdown of the fermenter due to overacidification must be prevented in advance. We proposed microorganisms to be monitored in order to gain insight into the development of methods to prevent overacidification. On the other hand, a comprehensive analysis of metabolites other than VFA should be performed to prevent overacidification and to further investigate the relationship with microbial population changes proposed in this study. The discovery of the candidate bacterial species for prediction of overacidification will help a development of testing approach, such as the daily quantification of specific species based on the isothermal amplification method (SmartAmp) with Exiton Primer [48,49].

## 4. Conclusions

In this study, we successfully performed overacidification and inhibition of methane production by a high substrate loading, in a batch culture of methanogenic microbial community with beverage waste. From the result of 16S rRNA amplicon sequencing analysis, it was considered that the predominant species *C. proteolyticus*, *D. tunisiensis*, *A*. *mobile*, and *T. oceani*, were involved in hydrolysis/acidogenesis/acetogenesis processes, and *M. soehngenii* was the major species conducting methanogenesis. Furthermore, monitoring the population changes of minor species revealed less than 0.2% of the total population represents the possibility of developing a method for prediction of overacidification occurrence. However, a research on the detailed relationship between the population change of the candidate indicator species and the amounts of various metabolites has been still remained. After the elucidation of the relationship, an adoption of low-cost, rapid, and easy-operating technique, such as SmartAmp, will enable us to predict overacidification occurrence on-site in methane fermentation plants.

## Figures and Tables

**Table 1:** Composition of batch cultures examined.

Run	SBWM (mL)	Sterilizaed Water (mL)	Inoculum (mL)
A	0	10.0	10.0
B	0.5	9.5	10.0
C	2.0	8.0	10.0
D	5.0	5.0	10.0
E	10.0	0	10.0

SBWM—simulant beverage waste mixture.

**Table 2:** The Simpson and Shannon indices of microbial communities in the family level.

Run	Simpson index			Shannon index		
	Day 5	Day 10	Day 15	Day 5	Day 10	Day 15
A	0.630	0.629	0.556	1.900	1.872	1.517
B	0.587	0.640	0.663	1.833	1.988	1.983
C	0.627	0.636	0.713	1.929	1.925	2.181
D	0.721	0.708	0.725	2.293	2.248	2.369
E	0.670	0.667	0.652	2.023	1.959	1.834

Simpson and Shannon indices of microbial community on day 0 were 0.757 and 2.545, respectively.

**Table 3:** Representation of the species revealed drastic changes in population in runs A, B, C, D, and E.

Culture Period (Day)	Run A				Run B				Run C				Run D				Run E			
	0	5	10	15	0	5	10	15	0	5	10	15	0	5	10	15	0	5	10	15
Species																				
*Candidatus Solibacter usitatus*	0.007	0.004	0.002	0.003	0.007	0.005	0.004	0.004	0.007	0.019	0.005	0.004	0.007	0.066	0.008	0.005	0.007	0.007	0.287	0.017
*Clostridium chauvoei*	VL	VL	VL	0.002	VL	0.263	0.185	0.073	VL	0.300	0.327	0.076	VL	1.982	0.802	0.634	VL	3.696	0.874	0.882
*Clostridium formicaceticum*	0.004	0.002	0.001	VL	0.004	VL	VL	0.001	0.004	0.004	0.001	0.001	0.004	0.017	0.003	0.006	0.004	VL	0.101	0.005
*Methanosaeta harundinacea*	0.256	0.186	0.168	0.122	0.256	0.183	0.338	0.434	0.256	0.153	0.269	0.586	0.256	0.119	0.145	0.202	0.256	0.050	0.037	0.020
*Methanothrix soehngenii*	0.945	0.635	0.422	0.001	0.945	0.566	1.164	1.358	0.945	0.517	0.947	2.126	0.945	0.364	0.442	0.734	0.945	0.147	0.110	0.072
*Neorickettsia helminthoeca*	0.027	0.031	0.019	0.028	0.027	0.061	0.052	0.080	0.027	0.137	0.157	0.143	0.027	0.002	0.376	0.291	0.027	0.002	0.001	VL
*Paludibacter propionicigenes*	ND	0.001	ND	0.002	ND	0.005	0.006	0.008	ND	0.027	0.032	0.038	ND	0.018	0.110	0.108	ND	ND	ND	0.109
*Petrimonas mucosa*	0.004	0.002	0.002	0.241	0.004	0.012	0.021	0.015	0.004	0.071	0.100	0.099	0.004	0.051	0.265	0.304	0.004	ND	VL	0.262

In all species identified in the amplicon sequence analysis, the species showing increases or decreases by more than 10-fold are listed and colored to represent the population range. Colors of population range (%): 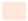 < 0.001; 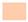 0.001–0.01; 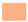 0.01–0.1; 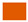 0.1–1.0; 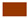 >1.0. The blank (white) panels indicate ‘not changed by more than 10-fold’ in the runs. ND and VL indicate ‘not detectable’ and ‘very low level less than 0.001%’, respectively.
